# Experiencing menopause in the UK: The interrelated narratives of normality, distress, and transformation

**DOI:** 10.1080/08952841.2018.1396783

**Published:** 2017-11-02

**Authors:** Isabel de Salis, Amanda Owen-Smith, Jenny L. Donovan, Debbie A. Lawlor

**Affiliations:** a School of Social and Community Medicine, University of Bristol, Bristol, UK; b MRC Integrative Epidemiology Unit at the University of Bristol, School of Social and Community Medicine, University of Bristol, Bristol, UK

**Keywords:** Idiom of distress, in-depth interviews, menopause, qualitative research, rite of passage, transformation, UK, women’s health

## Abstract

We investigated the experience and perspectives of menopause among 48 UK mothers through qualitative in-depth interviews. Interviews were analyzed thematically then explored using social science theories. Three interdependent narratives emerged: menopause as a normal, biological process, distinct from self and social transitions; menopause as struggle, an “idiom of distress” expressing upset, identity loss, shame, and social upheaval; and menopause as transformative and liberating, arising from biopsychic and relational changes. Some women followed a predictable “rite of passage” trajectory with transformation emerging from distress, but not all: Menopause arises from a complex interplay of personal predicament, somatic change, and sociocultural context.

## Introduction

The biomedical discourse of menopause is of “invariant biological transformation” (Lock & Kaufert, 2001) bringing decline and loss (Ferguson & Parry, 1998; Martin, 1993). But cross-cultural researchers like Lock argue that the somatic experience of menopause is never divorced from sociocultural processes nor personal stories and their sociopolitical contexts. Menopause is part of lived experience, needing to be understood as integral to life and not a discrete biological event. Meaning arises from the interaction between the biological and social where lived bodily experiences become social or visible to oneself and others (1993, 2010). Somatic experiences of menopause may vary cross-culturally (Beyene, 1986; Lock & Kaufert, 2001; Shea, 2006), socioeconomically (Dasgupta & Ray, 2013; Delanoe et al., 2012), ethnically (Nixon, Mansfield, Kittell, & Faulkner, 2001; Sampselle, Harris, Harlow, & Sowers, 2002; Wray, 2007), and across generations (Davis, 1997; Utz, 2011).

Recent research has explored the diversity of menopause for individual women, their “kaleidoscopic” contradictory emotions, and the uniqueness of each experience (Hvas & Gannik, 2008). The aim of this study was to explore the interaction between the biological and the social expressed in women’s menopausal experiences and perceptions and to understand both the uniqueness of each woman’s menopausal experience and the sociocultural tropes they expressed. We did so by looking at the interrelationship between somatic sensation, cultural imagery and life events, and the significance of menopause in the broader life context of women who are mothers living in the UK.

According to researchers in the UK, menopause can be experienced as a neutral, insignificant part of life (Wray, 2007), or women may neutralize menopause through “soldiering on” to distance themselves from a negative process associated with physical decline (Shore, 1999). Lock (1993) records that in Japan the correct way to deal with the disruptions of *kōnenki* (climacteric), part of the “natural life cycle,” was with endurance and appropriate attitude. Other events may overshadow menopause, such as illness and changes in family structure or employment (Ballard, Kuh, & Wadsworth, 2001), or the experience of menopause may be influenced by other midlife transitions (Dare 2011). On the other hand, menopause can be distressing with severe symptoms and feeling out of control (Nosek, Kennedy, & Gudmundsdottir, 2012). Menopause is reported to be negatively experienced where fertility status is highly valued, as in rural Iran (Khademi & Cooke, 2003).

Western women’s ambivalence to their changing bodies relates to negative cultural stereotypes of older women and valuing of youthful sexual attractiveness, while devaluing aging (Ballard, Elston, & Gabe, 2009; Banister, 1999; Hvas & Gannik, 2008; Rubinstein & Foster, 2013). The consequent stigmatization of menopause also relates to its medicalization (Ferguson & Parry, 1998), negative perceptions of somatic change, and silence surrounding it (Nosek, Kennedy, & Gudmundsdottir, 2010). Unsurprisingly then, psychosocial research reveals that how menopause is experienced may be linked to women’s self-esteem (Ayers, Forshaw, & Hunter, 2010; Hall, 2007; Kaufert, 1982).

However, women also report positive experiences of menopause (Gullette, 1997). For some Western women, aging can bring freedom, self-awareness, growth, reflection, and reassessment of role (Hvas & Gannik, 2008; Perz & Ussher, 2008). In parts of the world menopause has shown to be a time of freedom from previous restrictions (Beyene, 1986) and personal rebirth and empowerment (Morrison et al., 2014). Freed from menstrual uncleanliness, Iranian women are recorded as performing more religious rituals (Hakimi, Simbar, Ramezani Tehrani, Zaiery, & Khatami, 2016). Melby shows (2016) that when released from caring responsibilities, contemporary Japanese women anticipate a new life of self-actualization (*jibun-rashiku-ikiru*) through work or personal/social growth. Ballard et al.’s (2001) five stages describe a woman’s shifting relationship to her symptoms, while noting the importance of social context and status change. Dillaway’s (2005) American participants saw menopause as a return to prepubescence, more about reproductive status than aging. Nosek et al. (2012) followed one woman’s corporeal and spiritual transformation using Frank’s theory of chaos, restitution, and quest. Susan-Jane’s journey moved from night sweats and insomnia, treatment with Hormone Replacement Therapy (HRT), to finally the search for meaning. Mackie (1997) acknowledges the difficulty of experiencing menopause as transformative when menopause is considered loss and the multiple meanings of menopause subdued.

Anthropologists have observed the interrelationship between biological and social change, often drawing on Van Gennep’s (1977) and Turner’s (1991) studies of “rites of passage” or “life crisis” rituals. Despite his assertion that there are no known menopause rites, Van Gennep’s theory has been helpful for understanding menopause. Rasmussen (2000) shows how Tuareg women’s power was positively transformed on reaching postchildbearing years and their children reaching marriageable age. Kaufert (1982) notes that menopause was social renewal for Rajput women and social death for Fulani women. Both are rites of passage involving a socially recognized change of status within the kinship group: the “restructuring” of women’s kin relations (Turner, 1991). Diah (2014) uses the concept of liminality to describe urban Malays’ difficulties at menopause—a period of uncertainty and taboo with identities caught between traditional and Western ways—which they try to overcome with HRT. Skultans (1970) considers the climacteric is a rite of passage for Welsh women, though the absence of ritual means women lacked words to express their discontent. Less researched is contemporary UK women’s experience of menopause as a rite of passage relating to social or psychic change and how transformation relates to negative experiences of menopause.

The impact of fertility loss and changing kinship relations will likely be different for mothers and nonmothers. Despite most UK menopausal women having children, motherhood has not been prioritized in research. Exceptions are Utz’s (2011) different perspectives on aging between American mothers and daughters and Dillaway’s (2006) menopause studies of good mothering in the American Midwest. Motherhood roles may be ritually and socially redefined following fertility loss. In the shift from child-bearers to culture-bearers, Taureg women became metaphorical mothers (Rasmussen, 2000).

In this article, we report on three cultural narratives of menopause that reflect not only the diversity of experiences among women based in the UK but also the cultural tropes expressed simultaneously or successively in each woman going through menopause.

## Methods

We interviewed mothers selected from the Avon Longitudinal Study of Parents and Children (ALSPAC), a birth cohort that recruited over 14,000 pregnant women resident in southwest England between 1990 and 1992 (see http://www.bristol.ac.uk/alspac/). These mothers have completed questionnaires, brought their children to clinic assessments, and completed assessments themselves but have not previously been interviewed about motherhood, midlife, or menopause. A subset of 70 women was purposively sampled to include ranges of age, educational attainment (graduate/nongraduate), children’s ages, and menopausal stage defined according to the Stages of Reproductive Aging Workshop (STRAW) criteria (Harlow et al., 2012): premenopausal (preMP), menopausal (MP), and postmenopausal (PostMP).

We invited these women to be interviewed about their perceptions and experience of midlife. The ALSPAC Ethics and Law Committee gave ethical approval. One- to two-hour interviews loosely following a topic guide were audio-recorded usually in participants’ homes. The interviewer was female of similar age and personally interested in menopause, perhaps aiding a shared and reflective discussion. Women gave background information, including family structure and occupation. They were asked to explain their feelings about this time of their life, and they discussed changes in body, family and working life, help seeking, social support, ideas, and influences. Women had space to tell their own stories in their own time using their own terms. Thus we could gauge the relative significance of the biological and social processes of menopause, aging, and changes in family structures to women’s experience of midlife.

From the first three transcripts, IdeS and AOS identified themes to explore in subsequent interviews. This was further developed with JD working through four randomly extracted interviews from which we created an initial coding structure with @NVivo10 software. Extracts from the first 10 interviews were further discussed between all authors and academic colleagues. After coding we identified and explored themes grounded in the data and compared them across all interviews using constant comparison (Strauss, 1987). Although all authors readily agreed on themes in the initial stages of analysis, it became clear that individual accounts were not solely associated with specific themes: There was overlap requiring further analysis. We used matrices to compare summaries of key themes such as children leaving home, bodily change, relationship with partner, occupation, attitudes to aging, and menopause. We also created life stories of each woman to ensure that her biography was maintained. We found that women expressed nonlinear and contradictory views at different points in their interview, from which emerged the concept of narratives. We then investigated whether narratives were patterned according to age, menopausal status, social class, or family relationships.

## Results

A total of 48 women agreed to be interviewed during 2012–2013. The mothers (names changed) were aged between 49 and 56 years; 45 were white; 26 had degrees; five had vocational qualifications. Many were uncertain of their “clinical” menopausal stage and did not define this uniformly: Broadly, 16 women with few signs of menopause considered themselves premenopausal; 21 women with changing periods and other bodily sensations or experiences described themselves as menopausal or perimenopausal; and 11 women felt they were postmenopausal—their periods had long stopped, and sensations or experiences attributed to menopause had finished. Seventeen had a child ≤ 16 years, including five with a child at primary school. Three were grandmothers. Few women took HRT.

We found a spectrum of experiences from which we identified three distinct but overlapping narratives of menopause emerging from women’s accounts: menopause as a normal process, as a struggle provoking and expressing distress, and as a transformative experience. We use the term “narrative” for the cultural story or trope inherent in women’s experiences of their physical sensations, the interpretation of these sensations, and the social context in which they arise. During an interview, people might shift between narratives, either experiencing them side by side or successively. They may be expressed when women reflect backwards or anticipate the future. Women may express all three narratives, although often one dominated. Narratives were not exclusive, unrelated categories but interdependent reflections, commentaries or negations of each other. For example, the natural/normal and struggle/distress narratives were often perceived as mirror images. Here we use anthropological and sociological theories of chronic illness, transition states, and menopause to illuminate our findings. Children’s (C) and grandchildren’s (GC) ages are included for each woman. Unless stated otherwise, a woman considered her relationship with her partner to be unproblematic.

### Menopause: A normal process

In various ways women expressed the insignificance of menopause in their lives. Fertility loss felt inconsequential: They had “had their children.” Identity remained unaffected. Sarah (Chief-Operating-Officer.54 yrs.MP.C:18,21 yrs) expressed the relationship between bodily processes and identity:

I don’t think those physical aspects of your life define anything in life, actually… . It’s the other things, it’s your, you know, intellectual, personal and emotional development that defines who you are, not specific biological things.

Her body is experienced separately from self. Changing identity is related to other life transitions. After her children left home, Sarah described becoming her “own person again,” no longer “somebody’s wife, mother, daughter.” Her children departing had “felt very big” for Ruth (Psychotherapist.56 yrs.MP.C:20,22 yrs), who linked feeling “invisible and unattractive” to the empty house, not to menopause or marriage. For Bernadette (Home-maker.51 yrs.MP.C:12,19 yrs) with a younger child at home, neither menopause nor midlife were transitional: “It hasn’t been a traumatic change, no, or no big changes … a gentle evolution.”

Not overwhelmed by somatic sensations, these women could hold their sense of identity distinct from changing reproductive status and bodily processes. Some women barely noticed menopause and struggled to conceptualize symptoms that meant reifying a normal life process. Karena (Teacher.51 yrs.PostMP.C:12,15,18,20 yrs) said she “honestly hadn’t thought about it.” Menopause “just happens” without conscious engagement: It is natural and normal. Some actively held this image, drawing on experiences of childbirth to prove their bodies reliable and menopause similarly easy and natural, not “an illness” but an inevitable life process. Ruth explained:

It’s like childbirth, you know. Well it’s natural, it’s not like a terrible thing that strikes you, you know, it’s not an illness or, and you’ve got to just go through it in a way… . I’ve been very lucky as well, not been affected hardly at all.

Others relegated menopausal significance by choosing to experience sensations, generally unwanted and often called symptoms, as unproblematic. Polly (Health-visitor.53 yrs.MP.C:21,22,23 yrs) described her hot flushes as “nothing dramatic,” “mildly irritating,” “not a big issue.” “You just get on with it,” said Samantha (Nurse.52 yrs.PostMP.C:16,18,20 yrs). Compared to other women, she felt she had “got off pretty lightly.” Women contrasted their own experiences to those of family, friends, or colleagues and the image of menopause as distressing to render their own symptoms inconsequential: They were the lucky ones. Some learned strategies for rendering symptoms invisible or normal: “You don’t draw attention to yourself” (Pat.55 yrs.MP.C:15,18,21 yrs), “being positive helps” (Wendy. 50 yrs.MP.C:17,20 yrs) (Receptionists).

Some women actively resisted or willed the menopause not to happen, part of normalizing the process. Cherry (Mentor.54 yrs.PreMP.C:15,19 yrs) believed that, “You don’t have to just accept what’s coming.” To avoid becoming a “queen bitch” like her sister, she anticipated using diet and giving her body “a good talking to.” Caroline (Health-worker.52 yrs PreMP.C:18,20,22 yrs) jokingly declared, “I’m just determined not to have one.” Having a “high pain threshold,” she expected menopause to be “an easy experience” like childbirth. Like others, she invoked her mother’s unproblematic experience of menopause to determine her own: She “never made a big meal of it.” Medicines provided for few a powerful means for separating disease from self and shifting women away from a narrative of menopause as struggle. Sally (Art-teacher.51 yrs.MP.C:19,21 yrs) used HRT to reduce hot flushes, which were “absolutely foul,” waking her soaking wet and “not in control of my body,” poor sleep exacerbating her serious lung disease.

In women’s accounts of minimizing menopausal symptoms by not making “a drama,” “being positive” or not drawing “attention to oneself” lay an obligation to avoid performing it. Somatic symptoms should be controlled and invisible. Some sympathized with others’ unhappiness and potential stigmatization. Some censorious voices blamed individuals for being distressed: “You can predict people who’re going to have problems”; sisters’ “torrid times” with hot flushes, “dramatic” and “erratic” were anticipated responses to their personalities and “an excuse for bad behaviour.” Unlike her mother and herself, Kay (Ex-bank-clerk.54 yrs.PostMP.C:20 yrs), unemployed and single, felt women who “haven’t got enough things to think about” self-indulgently allow their symptoms to manifest. Either way the stigma of menopausal symptoms related to other women and not themselves.

Menopause either passed largely unnoticed or generated potentially uncontrollable, unwanted symptoms that some women actively resisted or normalized by comparing themselves favorably with others or downplaying their effect. As with chronic illness, these strategies helped preserve women’s identity intact (Bury, 1991). The moral imperative to hide menopausal symptoms is part of presenting the self as “culturally competent” (Bury, 2001), with the fear that incompetence is stigmatized (Nosek et al., 2010). It is part of the moral order where virtuous women “get on with it” (Stephens & Breheny, 2008). Like Japanese women who value endurance, the expression of symptoms can be a sign of personal weakness or self-indulgence (Lock, 1993). This was the most prevalent narrative. Menopause was objectified as a biological process separate from self, identity, and social transitions such as children leaving home, and from other difficult circumstances—unemployment, illness, loneliness, or relationship concerns (Ballard et al., 2001). Invoking their mother’s unproblematic experience helped some women imagine an easy menopause. Likening menopause to childbirth helped some women anticipate menopause as natural and inconsequential. Implicitly or explicitly women used the image of menopause as natural, normal, and inevitable, to contain symptoms, minimize its significance, and distance themselves from the narrative of menopause as struggle.

### The struggle of menopause

In this narrative distress at menopause was expressed in three interconnected ways: bodily changes; life changes; images of fertility loss and sexual decline. All aspects profoundly altered women’s sense of selfhood and identity. Clare (Civil-servant.55 yrs.MP.C:18,20 yrs) found menopause “horrible.” Changes such as croaky voice, globus [hystericus], weight gain, gray hair, sleeplessness, jumpiness, leg blisters, and waning libido profoundly altered her experience of herself:

I had awful, like blisters, appearing on my legs. I’ve never had that before, ridiculous. Really, really strange… . I just don’t feel like myself anymore really; you know I’m not the same shape, I don’t sound the same, you know my hair’s changed color. I just, you know I just, but then was I really me? … I don’t like feeling like this. I think I’d rather not feel like this; I’d rather feel you know like I did before.

Having no female kin to share experiences, Clare’s distress was worsened by the “taboo” that prevented people discussing menopause. Fear of ridicule deterred her from revisiting her doctor whom she felt dismissed her experiences: “You just feel like such a fool really.” Mandy’s (Labtechnician.55 yrs.MP.C:19,20 yrs) hormones affected her “whole outlook on … life.” She no longer knew who she was, how she felt, whether she and her husband were compatible. Mandy doubted that menopause was a socially acceptable explanation for her symptoms: Men “don’t want women using their hormones as an excuse”; her boss “might think, ‘Oh well, she’s past it then.’”

Jo (Healthworker.49 yrs.PreMP.C:11,13,17,19 yrs) described her novel “out of control” anger due to hormonal changes as “like a red mist feeling,” “an enormous wave,” “really strange.” Such uncontained emotional states challenged women’s sense of self both in the moment and over time. Hattie (Life-coach.53 yrs.MP.C:17,20 yrs) recently recovered after long illness, had wanted life as a “normal person” before becoming menopausal. Instead “the stress” in her body was sending her “a bit doolally” by throwing up “strange symptoms” of hot flushes, poor sleep, vaginitis, and stiffness. Her tense throat prevented her singing, making her feel “a bit weird and wonderful.” For the first time, situations such as presenting publicly made Hattie anxious, feel a loss of potency: “Feeling like an old person … this doesn’t feel like me.”

This time of life brings changes to family life. Distressing sensations were experienced within changing social relationships and circumstances. Mary (Carer.50 yrs.MP.C:17,20 yrs) attributed “getting wound up” to “the Change,” “exploding over silly things” such as a sink left full. She identified these frightening episodes as indicators of “rejection” by her husband and her boys leaving home, her biological and social worlds merging. As an explanation of her feelings, Mary said, “I live for my children.” In contrast, Mandy recognized two parallel processes. The distress of her children’s departure was distinct from but exacerbated by hormonal tearfulness. Cherry queried whether her daughter had taken her hormones with her when she left home.

For women identifying primarily with their child-rearing role, the change in status as children moved away brought identity crisis. They felt “redundant” or in a state of grief, regretting years lost to their teenagers’ chronic illnesses or risky behaviors. Jane (Teacher.53 yrs.PostMP.C:18,21,22 yrs) did not believe friends who claimed this was “the best time” of life. She tolerated disruptive hot flushes at work but struggled with the image of menopause as the “beginning of dying.” Her “degenerating body” was a “marker between still feeling reasonably youthful … and then suddenly not” and connected with the end of family life:

I’m [of] the Tracey Emin school of thought, who said it’s the beginning of dying, and I kind of think it is really because I am quite sort of family centric… . It’s an opportunity to go out and have more coffees with friends and things, but actually I’m quite sad about it as well and quite worried about this next stage of my life.

Life changes and menopausal imagery could create more distress than bodily sensations. Serena (HRconsultant.51 yrs.PostMP.C:21,21 yrs) enjoyed her hot flushes but was profoundly distressed by her daughters leaving, periods stopping, and imagining menopause was “the end”:

I feel like my life’s over, and it’s too late to start a new career, not that I want a new career, but it’s too late to attract men… . Beginning to get wrinkly and not being fertile anymore and not, not being needed as a mum, you know, the adoration. It marks the end of being young and attractive and fertile. And it’s a mark of the beginning of old age. I just feel like an old hag. That’s how I feel inside, so I just feel a has-been.

For some, this image of loss and ending was physically experienced: feeling “like an old hag” or “unsexy.” One of a few women who wanted more children, Tracey (Unemployed.49 yrs.MP.C:11,20,24,28 yrs), a divorced grandmother (GC:2,1½,1½yrs), felt this longing as a visceral agitation that had induced the menopause and was “actually killing me inside.” “This is going to be the end,” she said. Rosa (Lab-worker.54 yrs.MP.C:20,25 yrs) perceived the menopause as a midlife crisis and transition, interpreting her affair just before her impending infertility as a “biological” final bloom before a plant dies. For these women fertility was profoundly implicated in their sense of self; biological status was a marker of identity. Women talked of having “served one’s purpose” and daughters succeeding. Renata (Nurse.49 yrs.PostMP.C:20,22 yrs) cared for an elderly father and daughter with disabilities. Though her hot flushes and night sweats alongside demanding work made her “physically and mentally knackered,” the significance of menopause lay in waning fertility. Her identity, tethered to the corporeal, remained intact until her familiar menstrual cycle stopped:

I did, actually, mourn the passing of a phase of my life because I thought, this is a phase where you’re, kind of, fertile, and, you know, you’ve been doing this since you were 12, or whatever. You have your little cycle and then suddenly it’s not going to be happening. And it’s like, you know, it’s a bit of a realization that you are mortal, and things pack up, and you’ve reached this, sort of, your biological usefulness is over. You know what I mean? As a species, you can go now, you’ve done what you need to do, you’ve reproduced… . Like the old, sort of, withered apple on the tree, you know, there’s no seeds in it!

Menopause heralded the end of some women’s sex lives: Hormones keep you “sexy and young.” Menopause will be “a barren land” for Jo, putting “a kybosh” on revitalizing her inactive sex life with her husband. This conflation of fertility with sexuality appeared in other women’s accounts. Serena experienced the demise of fertility and ovulation as loss of maternal role and declining sexuality within a problematic marriage:

I felt like I gave out this vibe of not being sexy anymore, and I really do think there is a difference… . I think there must be something about women who are ovulating that’s, you know, subliminally attractive or attracts men.

Serena was “too ashamed” to tell her husband her periods had stopped because “it’s a mark of being old.” Jenny’s (Shopassistant.51 yrs.MP.C:14,20,21 yrs) menopause was suddenly induced by her “shocking” divorce. She wanted her periods because “hormones keep you young.” She embodied a prevalent image that around menopause husbands “go off and have an affair.”

Some women expressed this narrative alongside a narrative of menopause as natural and normal. Beth (Teachingassistant.55 yrs.MP.C:20,22 yrs) described wanting to live menopause as a natural process but at times felt herself descending into her dark “black dog” days and a deep grief for what she was losing: roles of mother and energetic teacher. In this narrative of menopause as struggle, the biological and social, the body and self become enmeshed: Menopause is implicated in the changing body, self, relational world, and social status. Distress provoked by shifts in family life was identified with the menopause through associated negative symptoms or created by and expressed within the image of menopause as loss, death, endings. Fertility loss was interpreted as the end of maternal and sexual roles and of declining attractiveness and youth. Such an image can be viscerally felt or even trigger the menopause. Here, hormones could “exit” following the shock of family members departing or immanent infertility.

The mirroring or resonance evident across social events, cultural images, and somatic sensations can range from a metaphorical (similarity) relationship, such as describing fertility loss in organic metaphors of a “barren land” or “withered apple tree” to a metonymic (contiguous) association whereby change in social status or images of loss were experienced somatically as powerful sensations that challenged selfhood and identity. Such associations in which bodily, sociocultural, and psychological processes mirror or resonate with each other are found in “idioms of distress.” Nichter (2008) suggests that idioms of distress provide a language for suffering that might not otherwise be fully expressed. As an image of ending and loss, menopause is a potent idiom straddling and reflecting these profound changes in the social and biological worlds of women: end of family life and maternal role; time disappearing to “sort oneself out” or find fulfilling work; feelings of impending mortality; incompatibility with partners; loss of attractiveness, femininity, and sexuality; end of ovulation and fertility. Menopause thus acts as a multivocal idiom of distress. This idiom was only partially legitimate to women themselves. The silence and stigma surrounding menopause was part of their distress.

### Transformation

Chronic illness is both “an assault on the person’s physical self” and “the person’s sense of identity” and future trajectories, what Bury termed “biographical disruption” (Bury, 1991). The resultant ruptures in relationships between body, self, and others may prompt a reexamination of core personal issues (Bury, 2001). Like chronic illness, menopause can disturb identities and disrupt biographies, with a consequent need to restructure identity and progress to the next stage. Mandy talked of needing to “grow up” and reject the world of “Jackie Magazine” (1970s teenage magazine) where “a problem … would all be sorted out if you met the right man and everybody would live happily ever after.” She referred to leg discomfort as “growing pains,” these somatic sensations mirroring her conscious reflections. Jane was “worried about the next stage of my life” after menopause, the end of family life and decline into aging. Caught in upsetting body changes and embodied images of dying and death, loss and endings, some women struggled to separate from previous roles.

However, gestating within some accounts of distress were references to “moving on” to a “next stage,” which might grow in emphasis during an interview. Tracey described fertility loss as viscerally killing her. Her daughter and grandchildren returning home forced her into a maternal role, rekindling her desire for more children. “Embracing” her hot flushes helped her accept the physical reality of fertility loss and “embrace the next step” of finding work or relationship and placing caring duties aside, moving on from a narrative of distress:

I’m just trying to enjoy the warm because I’m always cold… . So I’m trying to embrace something else to try and cope with the fact that I’ve lost the ability to have any more, and I’d like to have more and now I know I can’t, so I’ve upset myself now. So I suppose that’s why I’m trying to embrace the next step of my life, I suppose. I’m trying to force myself to embrace the fact that I’ve got to move on.

Serena also found hot flushes a source of warmth and nourishment, “nice and toasty,” after feeling cold all her life. During the interview, her account moved from the “trauma” of fertility loss experienced as “the end of an era”—aging, loss of maternal role, and declining sex life—to a reemerging sexuality reflecting a confident joyful self. Like others, she became aware of moving between narratives:

I’m happier in myself than I’ve ever been in my entire life, which probably sounds odd for you to hear given everything else I’ve been saying… . I’m happy about life and living and happy with the people I work with and happy with the friends I have and, and I think there’s a confidence that comes with age.

Since her periods ceased Serena, had been stuck in her “wilderness years” of feeling like “an old hag,” “unsexy,” and “not relevant anymore.” Overcoming her ageist notions, she was now entering “The Third Age.” Her unhappy marriage and shame at being menopausal transformed when she told her husband: “Since I’ve told him, maybe that’s why I’ve started feeling better about myself because he, he knows but, I, I’m beginning, I’m beginning to feel [laughs] sexier again now.”

Renata had previously associated fertility loss with the demise of sex, “everything’s packed up” and “fallen off” like the “withered apple tree.” But at the end of the interview she said:

When I got together with [partner], I just, sort of, to be quite, quite frank, I just said, “The shop’s shut, really, I don’t think anything works. I am ever so sorry.” He’s younger than me, as well, “So I can understand if you want to get your, sort of, exit ticket, I completely understand!” Anyway, bless his heart, he opened the shop, put the awnings out, the whole bloody array of everything on display, and oh my God, I’m having a wonderful time, I’ve come alive, blimey… . It’s bloody good, actually, yeah, I’m hot. Now, that bit doesn’t make me feel old!

Serena and Renata spontaneously experienced their sexuality reemerging after assuming it was dead. Moving from one narrative to another, these women followed a rite of passage trajectory (Van Gennep, 1977): Leaving behind a former [premenopausal] state, they entered a liminal period of separation before emerging into a new identity [postmenopausal]. Van Gennep’s three phases of “separation,” “transition,” and “incorporation” are reflected in women’s accounts: (a) separation from family members or shameful avoidance of discussing menopause; (b) menopause as an unknown process, a wilderness time of loss and endings marking a threshold, a “gateway” and “point in the sand”; and (c) liberation emerging from struggle and renewal of relationships. Rebirth following death matches Turner’s (1991) description of the transitional or “liminal” stage in the ritual process. It seems for such women that menopause can be usefully understood as a rite of passage despite not being ritualized.

One narrative might emerge from another, but narrative shifts were never entirely complete, and narratives may be held simultaneously. Despite Renata’s open upped sexuality, she later developed painful vaginitis. From the midst of a difficult menopause and mourning the departure of her eldest son, Hattie had “a new thought” about menopause in the days preceding, possibly inspired by feminist, anti-ageist ideas also evident in other women’s accounts. Her image of transformation—moving toward the unknown—ran alongside a different narrative:

Some people think of it, I think, that is quite, maybe, a useful way to think of it, as a process of transformation and moving on to something else. But most of the time it doesn’t feel like that, it feels like, what a pain… . It’s a kind of, oh bloody hell kind of thing!

In this narrative, menopause was the threshold between the concerns of pregnancy and motherhood and a state of liberation: a rebirth, not a time of distress. Like chronic illness, it could be “a form of disruption that can be turned into self-discovery and renewal” (Bury, 2001). According to Frank (1995), biographical disruption can present an opening for taking active responsibility for understanding our relationship to our self and others and identify our own personal values and sense of selfhood. This inner transformation of personal suffering accorded with some women’s experience: renegotiating kinship bonds to create a renewed sense of selfhood, finding freedom from or within the roles of wife, mother, grandmother. Menstruation ending was recognized as a significant biological event, “a good milestone,” for Rosa: liberation from pregnancy coinciding with “getting more comfortable in your own skin,” children grown up and a “good time in your career.” Kelly (Administrator.50 yrs.PreMP.C:21,22,23 yrs) described it “as another step along the long path of life” after attending school, growing up, getting married, and children becoming adults and moving on. Transformation was an inner psychic process arising from biological change and shifts in kinship relationships.

Most women experiencing menopause as transformation also expressed a narrative of loss and struggle. But not all. Becca (Retired-head-teacher.55 yrs.PostMP.C:13,17,20 yrs) adamantly stated that menopause had no significance for her: It “just switched off,” part of the “natural order of things.” Others “made it significant” by dramatizing their symptoms into illness. Later in the interview, she spontaneously reframed menopause. Whereas naturalness was part of making menopause normal and insignificant, the liberation from reproductive concerns alongside children leaving home and reduced workload made menopause a new beginning with her husband:

It’s the beginning of something new for me. And I think to some extent (Husband)’s gone through it himself a little bit, you know, whatever you call the male menopause… . It’s sort of liberating in that way… . I know there’s always an outside chance that you could fall pregnant but, the likelihood is I won’t, you know, so I’m looking to, or we are looking to you know, grow into each other again.

In her mid-40s Becca suffered a brain hemorrhage. This event forced her to “put things into perspective” and retire from her anxiety-making job because “actually I want to see my kids grow up.” Perhaps her illness acted as her distress narrative.

Julia (Healthworker.52 yrs.PreMP.C:10,15,21,25 yrs), who had a primary school child, created a conscious threshold between the “end of one stage of life … that’s being going on for years and years and years” and the next. Although a natural, biological process, she said it was also:

… kind of a gateway, isn’t it, into the next stage of your life? I mean, men don’t have that sort of definite closure point of one bit physically in the same way that women do… . Well, it will make you reflect, won’t it, on the 50 whatever years that you’ve had before because you won’t get 50 more years. So it kind of sets a point in the sand, doesn’t it, about how long you’ve been on the earth?

This narrative was the least common, affirming Mackie’s (1997) observation that the predominantly negative images of menopause suppress such experiences. Women did not have to suffer a difficult time to experience transformation, although most did: Menopause did not necessarily follow a predictable rite of passage trajectory composed of stages progressing in sequence from one to the next (Van Gennep, 1977). Instead, transformation seemed to arise from the complex and unpredictable relationship of cultural imagery, changing social status, and a biopsychic process unfolding from within. As Rappaport (2002) notes, ritual is a social process that imposes clear distinctions upon the continuum of nature and removes “psychic noise.” Without rituals recognizing status change, perhaps it is unsurprising that menopause follows an unpredictable and diverse course.

Shifts between narratives might be intentional. HRT stopped one woman’s overwhelming and distressing bodily sensations: She switched back from a narrative of distress to normality. Narratives might be held simultaneously with resultant tensions: Menopause was inevitable and natural on a good day, yet a struggle when suffering from “black dog” days or hot flushes. Menopause might be transformative on reflection, inspired by positive cultural imagery, but still “bloody hell” day to day. Although the shift between narratives of suffering to transformation often appeared in the interview as a sudden switch or instantaneous realization, it invariably followed a painful and overwhelming time of loss. In his illness narratives of chaos, restitution, and quest, Frank (1995) observes that transformation arises through reflecting on the suffering of chaos, discovering meaning within the pain, and finding a voice to say it. Women were often delightfully surprised in an interview when they expressed a transformative narrative: It may be the first time they retrospectively reflected on their changing lives. Women recounted overcoming the shame and taboo of menopause. Bodily changes took on metaphorical meaning such as “opening up” or “moving on.” Yet as Frank also notes, movement to a transformative narrative is never complete: Suffering lies within it. In a suffering narrative, the body-self is “unmade.” But “unmaking can be a generative process; what is unmade stands to be re-made” (1995, p. 172). Menopause might unmake women’s identities, but it can also be part of remaking them.

## Discussion

Three interrelated narratives of menopause emerged from the accounts of these mothers: menopause as a normal, biological process distinct from self, identity, and social transitions; menopause as a struggle provoking and expressing distress, a time of identity loss and social upheaval; and menopause as a time of liberation and transformation. The impact on identity and the relationship between cultural imagery, social change, and bodily experience were experienced differently in each narrative. This relationship between the social and biological gives menopause its significance (Lock & Nguyen, 2010).

These narratives of menopause were neither biologically nor socially determined but arose from a complex interaction of both (Lock & Nguyen, 2010). They did not occur/map onto distinct groups of women; menopausal stage was influential rather than determining. We found that women might express any of these narratives whether they had a child in primary school or in late teens or twenties, leaving home, or grandchildren. This study was mainly White women from southwest England of varying class backgrounds. Several studies found socioeconomic differences (Martin, 1993). For example, Delanoe et al. (2012), comparing French and Tunisian women living in both countries, found class more influential than nationality or geography. Less-educated West-Bengali women similarly fare worse (Dasgupta & Ray, 2013). We found no patterning of menopausal experience according to educational attainment or occupation. However, each woman’s socioeconomic background informed their unique experience of their body-selves. Whereas one unemployed single woman struggling with housing and work had no time for indulging in menopause, akin to Nixon et al’s (2001) rural African American women “staying strong,” another woman in similar circumstance found the image of fertility loss was “killing her” inside with the consequent need to “move on.”

The image of menopause as natural, normal, and inevitable allowed women to distance themselves from negative associations of menopause (Shore, 1999) and narratives of struggle. Accepting menopause as a natural process has been shown to help some Azeri-Iranian women cope (Hakimi et al., 2016). The objectification of menopause as separate from self does not just stem from Western dualist thinking and medicalization. Lock observed Japanese women also objectify the body to endure symptoms (Lock, 1993). Such objectification is also not necessarily part of the ongoing fragmentation of ourselves, a fear expressed by Martin (1993). As Frank (1995) astutely observed, body-self separation reflects acceptance of mortality and biological reality, not just denial of suffering.

Women might shift between narratives, either experiencing them simultaneously or successively, although often one dominated. Some women followed a rite of passage trajectory (Van Gennep, 1977) with transformation emerging from loss, shame, and struggle after a transitional period in the “wilderness.” For these women, the shift in narrative both signified and sprang from renegotiating kinship bonds (Turner, 1991), the unmaking and remaking of the body-self (Frank, 1995), and the restructuring of identity to create a renewed sense of selfhood (Banister, 1999). It might also be guided by liberatory cultural imagery and feminist-inspired emancipation. In Kaufert’s (1982) terms, the biological event of menopause was both a psychic as well as social rite of passage. Rappaport (2002) notes that ritual imposes a social structure over biological and psychic change. Perhaps this shift in narrative was not an inevitable linear progression for some women because menopause in the UK is neither ritualized nor associated with socially recognized status change. Instead, it is often silenced, shameful, and stigmatized.

The focus on the interrelationship between biology and culture found in some menopausal studies has also been expressed in feminist disability studies. Garland-Thomson (2002) suggests that disability is not defined solely by embodiment or environment but rather by the interactions between the two. She proposes “understanding disability as a pervasive cultural system that stigmatizes certain kinds of bodily variations” (2002, p. 5) through (negatively) interpreting and disciplining these bodily variations and creating an opposition between able-bodied and disabled bodies. She challenges the modernist idea of the embodied self as inherently stable instead of being perpetually metamorphosing. Inspired by this approach, we could understand menopause as a stigmatized bodily variation, part of a genderized cultural system that negatively pits the old, postmenopausal woman against the premenopausal beautiful, youthful, fertile woman. In this cultural system, menopausal bodies are both failing and unstable with out of control, declining hormones. We could challenge the normativity of menopause as one embodied process of universal loss and decline, rather than multiple embodied processes. A feminist disability theory includes lived experiences as well as representations, highlighting complexities rather than resorting to “ideological policing.” So we can see HRT as both normalizing the abnormal woman with her out of control body and yet also allowing her to function and live her life.

Some women’s experiences challenge the dominant ethos of the genderized and dichotomized value system either through ignoring or rejecting negative imagery or more defiantly by seeing the menopausal transition as transformative and liberating. Yet these narratives could also be informed by a rising cultural discourse promoting positive aging in opposition to old age as decline, noted by researchers in aging studies, where sexiness and being sexually active are important indicators of not being old (Marshall, 2011). Sandberg (2015) states that the sexualities of older adults are now pitted against an earlier discourse of the “asexual oldie” where older bodies, especially women’s, are both undesiring and undesirable. Yet what may initially be positive and affirmative—“the sexy senior”—may become a new tyranny of later-life sexuality where older adults must stay sexually active. Marshall (2011) states that this new disciplinary technology of sexiness based on an opposition between the sexy and asexual older adult perpetuates a decontextualized and heteronormative notion of aging, one that promotes a narrow conception of successful aging in gendered and heterosexist ways. Furthermore, it denies the actual complexities of later-life sexuality and bodily changes such as vaginal dryness.

Much of the liberatory narrative in this article relates to renewed sexuality within a heteronormative frame of sexiness, fertility, and successful aging. So it’s useful to hold a cautionary voice lest such a narrative becomes a tyrannical expectation of how women should go through menopause. However, these women’s transformative experiences arose from the need to restructure identity. Holding a positive and liberating narrative helped them through difficult times and offered an alternative to derogatory voices of menopause. A transformative narrative can both promote heteronormative notions of successful aging while also challenging a genderized value system that negatively defines menopause.

Our results are based on a sample of mainly White women, and future research will be more ethnically diverse. Our findings are based on one interview per woman. Subsequent interviews with these women will help inform our ideas of menopause being a transformative event. All the women in our study were mothers and had been active participants in a cohort study. Our findings may have been different if our sample included childless women without such a commonality. We aim to focus on childless women in further research.

## Conclusion

We identified three narratives of menopause emerging from women’s accounts: normality, distress, and transformation. Some women followed a “rite of passage” trajectory, but this progression was neither inevitable, complete, nor determining. These findings revealed a complex and sometimes incomplete process of movement between narratives, different to linear accounts of the menopausal journey (Ballard et al., 2001; Nosek et al., 2012). Such varied yet shared experiences suggest that any one woman’s menopause should not be expected to follow a particular course: Her experiences arise from a complex interplay of personal predicament, somatic change, and sociocultural context. These findings accord with current research emphasizing the diversity of menopausal experience (Banister, 1999; Hvas & Gannik, 2008). Yet it is also helpful to see the commonalities. Conceptualizing our findings as narratives allows concomitant experiences to be seen as simultaneous rather than contradictory. Perceiving menopause as natural and inevitable allowed women to distance themselves from menopause. Holding a positive and liberating narrative helped women through difficult times and offered an alternative to derogatory voices of menopause. By acknowledging different women’s experiences and the cultural narratives they express, we can develop alternative, more liberating images of menopause.

## References

[CIT0001] Ayers B., Forshaw M., & Hunter M. S. (2010). The impact of attitudes towards the menopause on women’s symptom experience: A systematic review. *Maturitas*, 65(1), 28–36. doi:10.1016/j.maturitas.2009.10.016 19954900

[CIT0002] Ballard K. D., Elston M. A., & Gabe J. (2009). Private and public ageing in the UK the transition through the menopause. *Current Sociology*, 57(2), 269–290. doi:10.1177/0011392108099166

[CIT0003] Ballard K. D., Kuh D. J., & Wadsworth M. E. J. (2001). The role of the menopause in women’s experiences of the “change of life.” *Sociology of Health & Illness*, 23(4), 397–424. doi:10.1111/1467-9566.00258

[CIT0004] Banister E. M. (1999). Women’s midlife experience of their changing bodies. *Qualitative Health Research*, 9(4), 520–537. doi:10.1177/104973299129122045

[CIT0005] Beyene Y. (1986). Cultural significance and physiological manifestations of menopause. A biocultural analysis. *Culture, Medicine and Psychiatry*, 10(1), 47–71. doi:10.1007/BF00053262 3698649

[CIT0006] Bury M. (1991). The sociology of chronic illness—A review of research and prospects. *Sociology of Health & Illness*, 13(4), 451–468. doi:10.1111/j.1467-9566.1991.tb00522.x

[CIT0007] Bury M. (2001). Illness narratives: Fact or fiction? *Sociology of Health & Illness*, 23(3), 263–285. doi:10.1111/shil.2001.23.issue-3

[CIT0008] Dare, J. S. (2011). Transitions in midlife women’s lives: Contemporary experiences. *Health Care for Women International*, 32(2), 111–133. 21229427 10.1080/07399332.2010.500753

[CIT0009] Dasgupta D., & Ray S. (2013). Attitude toward menopause and aging: A study on postmenopausal women of West Bengal. *Journal of Women & Aging*, 25(1), 66–79. doi:10.1080/08952841.2012.720203 23199313

[CIT0010] Davis D. L. (1997). Blood and nerves revisited: Menopause and the privatization of the body in a Newfoundland postindustrial fishery. *Medical Anthropology Quarterly*, 11(1), 3–20. doi:10.1525/maq.1997.11.issue-1 9138768

[CIT0011] Delanoe D., Hajri S., Bachelot A., Draoui D. M., Hassoun D., Marsicano E., & Ringa V. (2012). Class, gender and culture in the experience of menopause. A comparative survey in Tunisia and France. *Social Science & Medicine*, 75(2), 401–409. doi:10.1016/j.socscimed.2012.02.051 22575696

[CIT0012] Diah M. (2014). *Liminality and menopause: The urban Malay women’s persepctive* In Karim A. & Diah M. N. (Eds.), *Traditionalism and modernity: Issues and perspectives in sociology and social anthropology* (pp. 77–97). Singapore: Partridge.

[CIT0013] Dillaway H. E. (2005). Menopause is the “good old”—Women’s thoughts about reproductive aging. *Gender & Society*, 19(3), 398–417. doi:10.1177/0891243204271350

[CIT0014] Dillaway H. E. (2006). Good mothers never wane: Mothering at menopause. *Journal of Women and Aging*, 18(2), 41–53. doi:10.1300/J074v18n02_04 16782659

[CIT0015] Ferguson S. J., & Parry C. (1998). Rewriting menopause: Challenging the medical paradigm to reflect menopausal women’s experiences. *Frontiers—A Journal of Women Studies*, 19(1), 20–41. doi:10.2307/3347130

[CIT0016] Frank A. (1995). *The wounded storyteller: Body, illness, and ethics*. Chicago, IL: University of Chicago Press.

[CIT0017] Garland-Thomson R. (2002). Integrating disability, transforming feminist theory. *NWSA Journal*, 14(3), 1–32. doi:10.2979/nws.2002.14.issue-3

[CIT0018] Gullette M. (1997). Menopause as magic marker: Discursive consolidation in the United States, and strategies for cultural combat. In Komesaroff P., Rothfield P., & Daly J. (Eds.), *Reinterpreting menopause: Cultural and philosophical issues* (pp. 176–199). New York, NY: Routledge.

[CIT0019] Hakimi S., Simbar M., Ramezani Tehrani F., Zaiery F., & Khatami S. (2016). Women’s perspectives toward menopause: A phenomenological study in Iran. *Journal of Women & Aging*, 28(1), 80–89. doi:10.1080/08952841.2014.954502 26735699

[CIT0020] Hall R. (2007). Menopause: A biocultural perspective. *American Journal of Human Biology*, 19(3), 451–453. doi:10.1002/ajhb.20652

[CIT0021] Harlow S. D., Gass M., Hall J. E., Lobo R., Maki P., Rebar R. W., … de Villiers T. J. (2012). Executive summary of the Stages of Reproductive Aging Workshop+10: Addressing the unfinished agenda of staging reproductive aging. *Climacteric*, 15(2), 105–114. doi:10.3109/13697137.2011.650656 22338612 PMC3580996

[CIT0022] Hvas L., & Gannik D. E. (2008). Discourses on menopause—Part II: How do women talk about menopause? *Health*, 12(2), 177–192. doi:10.1177/1363459308091428 18400828

[CIT0023] Kaufert P. A. (1982). Anthropology and the menopause—The development of a theoretical framework. *Maturitas*, 4(3), 181–193. doi:10.1016/0378-5122(82)90048-2 7154971

[CIT0024] Khademi S., & Cooke M. S. (2003). Comparing the attitudes of urban and rural Iranian women toward menopause. *Maturitas*, 46(2), 113–121. doi:10.1016/S0378-5122(03)00182-8 14559382

[CIT0025] Lock M. (1993). *Encounters with aging: Mythologies of menopause in Japan and North America*. Berkeley, CA: University of California Press.

[CIT0026] Lock M., & Kaufert P. (2001). Menopause, local biologies, and cultures of aging. *American Journal of Human Biology*, 13(4), 494–504. doi:10.1002/(ISSN)1520-6300 11400220

[CIT0027] Lock M., & Nguyen V.-K. (2010). *An anthropology of biomedicine*. Chichester, UK: Wiley-Blackwell.

[CIT0028] Mackie F. (1997). The left hand of the goddess: The silencing of menopause as a bodily experience of transition In Komesaroff P., Rothfield P., & Daly J. (Eds.), *Reinterpreting menopause: Cultural and philosophical issues* (pp. 17–31). New York, NY/London, UK: Routledge.

[CIT0029] Marshall B. L. (2011). The graying of “sexual health”: A critical research agenda. *Canadian Review of Sociology*, 48(4), 390–413. doi:10.1111/j.1755-618X.2011.01270.x 22400206

[CIT0030] Martin E. (1993). *The woman in the body: A cultural analysis of reproduction*. Milton Keynes, UK: Open University Press.

[CIT0031] Melby M. K. (2016). Lessons on aging: Hopes and concerns of Japanese women at midlife. *Journal of Women & Aging*, 28(2), 127–140. doi:10.1080/08952841.2014.950544 26697746

[CIT0032] Morrison L. A., Brown D. E., Sievert L. L., Reza A., Rahberg N., Mills P., & Goodloe A. (2014). Voices from the Hilo Women’s Health Study: Talking story about menopause. *Health Care for Women International*, 35(5), 529–548. doi:10.1080/07399332.2013.829067 24134306 PMC3925469

[CIT0033] Nichter M. (2008). Coming to our senses: Appreciating the sensorial in medical anthropology. *Transcultural Psychiatry*, 45(2), 163–197. doi:10.1177/1363461508089764 18562492

[CIT0034] Nixon E., Mansfield P. K., Kittell L. A., & Faulkner S. L. (2001). “Staying strong”: How low-income rural African American women manage their menopausal changes. *Women & Health*, 34(2), 81–95. doi:10.1300/J013v34n02_06

[CIT0035] Nosek M., Kennedy H. P., & Gudmundsdottir M. (2010). Silence, stigma, and shame a postmodern analysis of distress during menopause. *Advances in Nursing Science*, 33(3), E24–E36. doi:10.1097/ANS.0b013e3181eb41e8 20693828

[CIT0036] Nosek M., Kennedy H. P., & Gudmundsdottir M. (2012). “Chaos, restitution and quest”: One woman’s journey through menopause. *Sociology of Health & Illness*, 34(7), 994–1009. doi:10.1111/j.1467-9566.2011.01453.x 22471763

[CIT0037] Perz J., & Ussher J. M. (2008). “The horror of this living decay”: Women’s negotiation and resistance of medical discourses around menopause and midlife. *Womens Studies International Forum*, 31(4), 293–299. doi:10.1016/j.wsif.2008.05.003

[CIT0038] Rappaport R. (2002). *Ritual and religion in the making of humanity*. Cambridge, UK: Cambridge Univeristy Press.

[CIT0039] Rasmussen S. J. (2000). From childbearers to culture-bearers: Transition to postchildbearing among Tuareg women. *Medical Anthropology*, 19(1), 91–116. doi:10.1080/01459740.2000.9966170 11833972

[CIT0040] Rubinstein H. R., & Foster J. L. H. (2013). “I don’t know whether it is to do with age or to do with hormones and whether it is do with a stage in your life”: Making sense of menopause and the body. *Journal of Health Psychology*, 18(2), 292–307. doi:10.1177/1359105312454040 22904151

[CIT0041] Sampselle C., Harris V., Harlow S., & Sowers M. (2002). Midlife development and menopause in African American and Caucasian women. *Health Care for Women International*, 23(4), 351–363. doi:10.1080/0739933029008928 12148913

[CIT0042] Sandberg L. (2015). Sex, sexuality and later life In M W. & Twigg J. (Ed.), *Routledge handbook of cultural gerontology* (pp. 218–225). London, UK: Routledge.

[CIT0043] Shea J. L. (2006). Cross-cultural comparison of women’s midlife symptom-reporting: A China study. *Culture, Medicine and Psychiatry*, 30(3), 331–362. doi:10.1007/s11013-006-9020-4 17048096

[CIT0044] Shore G. (1999). Soldiering on: An exploration into women’s perceptions and experiences of menopause. *Feminism & Psychology*, 9(2), 168–178. doi:10.1177/0959353599009002009

[CIT0045] Skultans V. (1970). Symbolic significance of menstruation and menopause. *Man*, 5(4), 639–651. doi:10.2307/2799108

[CIT0046] Stephens C., & Breheny M. (2008). Menopause and the virtuous woman: The importance of the moral order in accounting for medical decision making. *Health*, 12(1), 7–24. doi:10.1177/1363459307083695 18073244

[CIT0047] Strauss A. (1987). *Qualitative analysis for social scientists*. New York, NY: Cambridge University Press.

[CIT0048] Turner V. (1991). *The ritual process: Structure and anti-structure*. New York, NY: Cornell University Press.

[CIT0049] Utz R. L. (2011). Like mother, (not) like daughter: The social construction of menopause and aging. *Journal of Aging Studies*, 25(2), 143–154. doi:10.1016/j.jaging.2010.08.019

[CIT0050] Van Gennep A. (1977). *The rites of passage*. London, UK: Routledge & Kegan Paul.

[CIT0051] Wray S. (2007). Women making sense of midlife: Ethnic and cultural diversity. *Journal of Aging Studies*, 21(1), 31–42. doi:10.1016/j.jaging.2006.03.001

